# Effect of the Presence of Emergency Departments With 300 or More Hospital Beds in Health Service Areas on 30-Day Mortality in Korea: A Nationwide Retrospective Cross-sectional Study

**DOI:** 10.34172/ijhpm.2024.8010

**Published:** 2024-05-12

**Authors:** Stephen Gyung Won Lee, Haibin Bai, Joo Won Park, Seonhwa Lee, Mi Young Kwak, Won Mo Jang

**Affiliations:** ^1^Department of Emergency Medicine, Seoul Metropolitan Government-Seoul National University Boramae Medical Center, Seoul, South Korea.; ^2^Division of General Internal Medicine, Section of Biomedical Informatics and Data Science, School of Medicine, Johns Hopkins University, Baltimore, MD, USA.; ^3^Center for Public Healthcare, National Medical Center, Seoul, South Korea.; ^4^Department of Public Health and Community Medicine, Seoul Metropolitan Government-Seoul National University Boramae Medical Center, Seoul, South Korea.; ^5^Department of Health Policy and Management, Seoul National University College of Medicine, Seoul, South Korea.

**Keywords:** Emergency Department, Health Services Accessibility, Healthcare Disparities, Health Services Administration, Health Service Area, Mortality

## Abstract

**Background:** Disparities in emergency care accessibility exist between health service areas (HSAs). There is limited evidence on whether the presence of an emergency department (ED) that exceeds a certain hospital bed capacity is associated with emergency patient outcomes at the regional level. The objective of this study was to evaluate the effect of HSAs with or without of regional or local emergency centers with 300 or more hospital beds (EC300 or nEC300, respectively) by comparing the 30-day mortality of patients with severe emergency diseases (SEDs) admitted to the hospital through the ED.

**Methods:** The study retrospectively evaluated data from the National Health Information Database (NHID) of the National Health Insurance Service (NHIS) Claims database and enrolled patients who were admitted from the ED for SEDs. SEDs were defined using ICD-10 (International Classification of Diseases 10th Revision) codes for 28 disease categories with high severity, and 56 HSAs were designated as published by the NHIS. We performed hierarchical logistic regression analysis using multilevel models with the generalized linear mixed model (GLIMMIX) procedure to evaluate whether EC300 was associated with the 30-day mortality of SED patients, adjusting for patient-level, prehospital-level, hospital-level, and HSA-level variables.

**Results:** In total, 662 478 patients were analyzed, of whom 54 839 (8.3%) died within 30 days after hospital discharge. Of the 56 HSAs, 46 (82.1%) were included in the EC300 group. After adjustment for patient-level, prehospital-level, hospital-level, and HSA-level variables, nEC300 was significantly associated with increased 30-day mortality in SED patients (adjusted odds ratio [AOR]: 1.33, 95% CI: 1.137-1.153). In addition, patients who visited EDs with fewer annual SED admissions were associated with higher 30-day mortality.

**Conclusion:** nEC300 had a greater risk of 30-day mortality in patients treated with SEDs than EC300. The results indicate that not only the number of EDs in each HSA is important for ensuring adequate patient outcomes but also the presence of EDs with adequate receiving capacity.

## Background

**Key Messages**

**Implications for policy makers**

- There have been numerous efforts to improve the accessibility of essential care, including emergency care, using an increasing number of hospitals in specific health service areas (HSAs).
- This study revealed that both the number of hospitals with an emergency department (ED) and the existence of a hospital with an ED that exceeds a certain hospital bed capacity threshold in an HSA has a positive association with patient outcomes.
- A health services area having a hospital with more than 300 beds had lower 30-day mortality than did an area without such beds after adjusting for patient, prehospital, hospital, and area factors.
- A hospital bed count threshold of 300 can be helpful for reconfiguring the regional emergency care delivery system to counteract accessible disparities in South Korea.

**Implications for the public**

Depending on where they live, the difference in essential care outcomes, including emergency care, makes people living in higher-mortality areas aware of inequalities in medical care. We evaluated the association between the presence of a hospital with more than 300 beds with an emergency department (ED) in the health service area (HSA) and the 30-day mortality rate of patients with severe emergency conditions. Our results indicated that the mortality rate in areas with a hospital with more than 300 beds was lower than that in areas without one. In South Korea, the existence of a hospital with more than 300 beds can be a helpful criterion when evaluating the emergency medical responsibility capacity of a place where people live.

Timely access to emergency care is crucial for improving patient care outcomes, especially for patients with time-sensitive diseases.^1-3^ However, there are regional disparities in medical resources and emergency care accessibility that are associated with regional differences in patient outcomes such as mortality.^4-6^ Thus, a systematic strategy is needed to establish an efficient nationwide emergency care system that promotes patient accessibility to emergency departments (EDs).

 One strategy to improve ED accessibility is to ensure that EDs are located in appropriate areas.^7^ Since the location of healthcare institutions depends not only on patient emergency care needs but also on economic and geographic factors, to promote healthcare accessibility policies and monitor regional emergency medical resources, the South Korean government and researchers have designated 56 health service areas (HSAs). Regarding regional disparities in emergency care, the Korean government maintains a policy that supports the operating expenses of emergency medical institutions in vulnerable areas with emergency medical funds. Because there is no legal basis for compelling the establishment of EDs for each HSA, disparities in emergency medical resources exist between the HSAs despite government support, resulting in discrepancies in emergency care accessibility. In comparison, the United States government has implemented policies to mitigate this discrepancy by introducing rural emergency hospitals and critical access hospitals to rural areas,^8,9^ whereas in Shanghai, the health-care reform policy has taken measures to reallocate healthcare resources to improve healthcare accessibility in rural areas.^10^

 To reduce this gap, in 2019 the South Korean government formulated a national-level reformation plan for the emergency care system that included specific strategies for expanding essential emergency care resources, educating healthcare providers, improving the accountability of local hospitals and strengthening networks between hospitals.^11^ In 2021, the government published a follow-up plan to achieve “sufficient regionalization of emergency medical care” by increasing ED accessibility across HSAs. The policy aims to reform the ED level classification, redesignate existing EDs to each new level, and regulate the roles of each ED level that have previously been categorized into four levels—regional emergency medical centers, local emergency medical centers, local emergency medical agencies and unqualified emergency agencies—by the Emergency Medical Service Act. Legal standards that focus on ED resources are required for EDs to be designated to each ED level. The regional emergency medical center is the highest-level ED, and as of 2021 there were 38 regional emergency medical centers, 128 local emergency medical centers, and 238 local emergency medical agencies legally registered in Korea that serve a population of approximately 50 million inhabitants living in an area of 100 210 km^2^.

 To reform the emergency medical system in Korea according to policy initiatives and to allocate appropriate medical resources across HSAs, a minimum hospital bed number threshold is required, in addition to setting the minimum number of tertiary referral hospitals required per HSA to ensure adequate patient outcomes, as previous studies have reported that higher hospital volume is associated with positive outcomes.^12-16^ Although the cutoff value of 300 hospital beds is generally accepted as the standard in Korean healthcare and has been set as the legal standard for designating regional emergency centers and classifying general hospitals,^17^ and because previous studies have evaluated the relationship between hospital bed count and mortality,^18-21^ no studies have evaluated the 300-hospital-bed cutoff value for HSA-level analysis. We defined the presence of regional or local emergency centers with 300 or more hospital beds as EC300. We hypothesized that HSAs with EC300 would have lower 30-day mortality from severe emergency diseases (SEDs) admitted to the hospital through the ED. The objective of this study was to evaluate the effect of HSAs with EC300 or without EC300 (nEC300) on the 30-day mortality of patients with SEDs admitted to the hospital through the ED.

## Methods

###  Study Design and Setting

 This cross-sectional study retrospectively evaluated data from the National Health Information Database (NHID) of the National Health Insurance Service (NHIS). The National Health Insurance (NHI) program has universal coverage of 97% of the Korean population, which accounts for approximately 50 million people. All claims covered by the NHI program were collected from the NHID. The NHID includes patient demographic variables and records on inpatient and outpatient healthcare usage, such as diagnosis, hospital length of stay, prescription records and date of death. In addition, the NHID collects information regarding healthcare institutions, such as the level of the healthcare institution, location, number of staff and equipment.^22^ Using personal identification codes collected by the NHID, we were able to collect patient-level, prehospital-level, hospital-level, and HSA-level variables. Patients, individual institutions, and HSAs were divided into three clusters according to the characteristics of the index. In addition, individual institutions were classified into prehospital and hospital levels. Patient-level data included sex, age and Charlson comorbidity index (CCI), while prehospital-level and hospital-level data included type of access to the ED, travel time to the ED, annual number of SED patients who visited the ED and level of ED variables. Finally, the HSA level includes the relevance index, the number of regional and local emergency medical centers (per 100 000 population) and the number of emergency medical centers with 300 or more beds within the HSA. To compare the effects on 30-day mortality, variables were organized and analyzed for each level of patient characteristics (Supplementary file 1, Table S1).

###  Study Population

 The study enrolled patients who were registered in the NHID and admitted from the ED for SED between January 2016 and December 2016. SED was defined using ICD-10 (International Classification of Diseases 10th Revision) codes of 28 disease categories with high severity designated and monitored for hospital ED performance by the Korean Ministry of Health and Welfare (Table S2).^23,24^ We selected 6 307 363 episodes that visited the ED for SED and were admitted to medical facilities from January 2016 to December 2016. We excluded hospitalization episodes in oriental medicine institutions, dental medical institutions, police hospitals, veterans hospitals, psychiatric hospitals, and rehabilitation hospitals. This left us with 762 381 eligible episodes. Next, we excluded episodes that did not have a hospital or home address. The final number of episodes for the current study was 662 478. We treated a single admission episode as a case where a patient stayed in the same hospital for one day and left on the same day. This was because a hospital could submit multiple claims for one day if they were separated by monthly periods. Due to the nature of the study methodology, which used claims data of NHID, a patient could be enrolled in the analysis multiple times if the patient was admitted multiple times for SED during the study period.

###  Exposure

 The main focus of the study was whether a regional or local emergency center with 300 or more hospital beds was present in each HSA (EC300 vs. nEC300). We designated a cutoff of 300 hospital beds as the generally accepted standard in Korean healthcare. A total of 56 HSAs were designated in Korea as described in a previous study by the NHIS Service.^25^

###  Outcome Measures

 The primary outcome of the study was mortality within 30 days of discharge (30-day mortality), which included in-hospital mortality.

###  Statistical Analysis

 Variables are reported as number (percentage) and were compared using the chi-squared test or Fisher’s exact test. We performed hierarchical logistic regression analysis using multilevel models with the generalized linear mixed model (GLIMMIX) procedure to evaluate whether EC300 was associated with 30-day mortality. The mortality ratio was calculated by transforming the Summary Hospital-level Mortality Indicator (SHMI) (UK Health & Social Care Information Center ) according to the Korean context. SHMI is the ratio of actual deaths to expected deaths. The expected number of deaths in this study was calculated by correcting for age, sex, hospitalization route, diagnostic group, income quartile, CCI, and hospitalization type.^37^ CCI is an assessment tool designed to predict mortality by classifying comorbidities.^38^ In this study, the companion disease score was calculated using all the injuries recorded in individuals in the NIH claim-based data for one year. A higher CCI means higher mortality and higher resource utilization, and different versions of CCI include different diseases and disease weights. In this study, accompanying disease scores were calculated based on the revised CCI version of Dr. Foster Intelligence used in the UK SHMI calculation (Table S3). The adjusted odds ratios (AORs) and 95% confidence intervals (CIs) were calculated for the outcomes after adjusting for patient-level, prehospital-level, hospital-level and HSA-level variables. The hospital-level variables analyzed were the annual volume of SED patients visiting the hospital’s ED and the level of ED (regional emergency center, local emergency center with 300 or more hospital beds, local emergency center with fewer than 300 hospital beds, local emergency agency, and unqualified emergency agency) classified according to the Emergency Medical Service Act. The HSA-related variables included the Relevance Index,^26^ the number of regional and local emergency medical centers per 100 000 people, and EC300. The relevance index refers to the percentage of residents’ medical service utilization in a region relative to their total medical service utilization. All the adjusted variables are listed within the predefined categories in Table S1. All tests were two-tailed, and *P* < .05 were considered statistically significant. All statistical analyses were performed using SAS software (version 9.4; SAS Institute, Inc., Cary, NC, USA).

## Results

**Figure 1:**
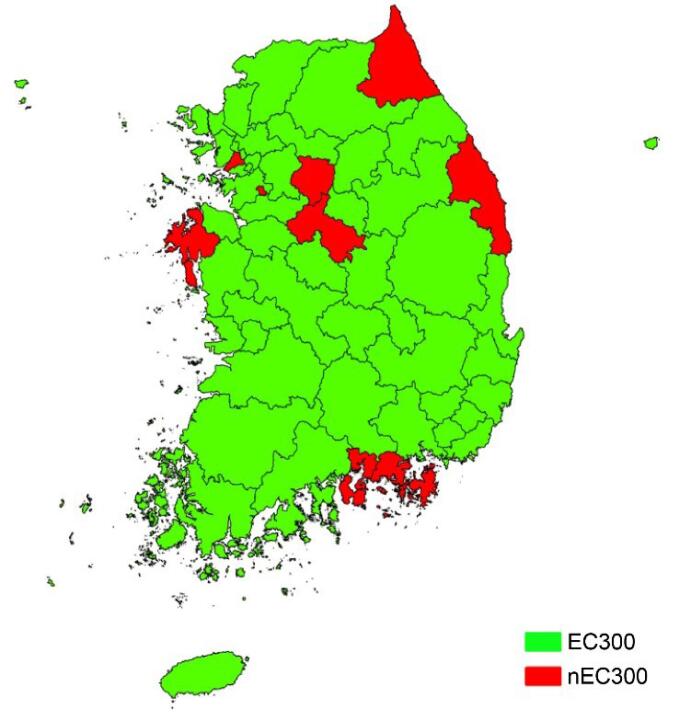
Distribution of EC300 and nEC300 Across 56 Health Service Areas. EC300, regional or local emergency center with 300 or more hospital beds located within an HSA; nEC300, no regional or local emergency center with 300 or more hospital beds within an HSA. Abbreviation: HSA, health service area.

 Among the 662 478 patients eligible for analysis, 340 314 patients (51.4%) were male, and 272 387 (41.1%) were aged 65 or older. In total, 54 839 patients (8.3%) died within 30 days after hospital discharge. Of the 56 HSAs, 46 (82.1%) were included in the EC300 cohort. The distributions of EC300 and nEC300 over HSAs are displayed in Figure 1. The number of regional or local emergency centers with 300 or more hospital beds ranged from 0 to 31 centers across the HSAs (Table S4).

###  Baseline Characteristics

 The baseline characteristics of the whole study population according to 30-day mortality are displayed in Table 1. According to the baseline analysis, male sex, older age, CCI, and longer travel time from the scene to the ED were associated with greater mortality. Patients in local emergency centers with fewer than 300 hospital beds had significantly greater mortality than those in other EDs with higher hospital beds. Higher mortality was observed in HSAs with fewer regional and local emergency medical centers. Compared to that of the EC300, the mortality of the nEC300 was greater (8.0 vs. 12.2, *P* < .0001). The distributions of primary diagnoses according to study group and the distributions of primary diagnoses causing mortality according to study group are displayed in Tables S5 and S6.

**Table 1:** Baseline Characteristics of the Study Population According to Survival Status

	**Survived (N = 607639)**		**Died (N = 54839)**		***P*** ** Value**
	**No.**	**%**	**No.**	**%**	
**Patient Level**					
Gender					
Male	307677	90.4	32637	9.6	<.0001
Female	299962	93.1	22202	6.9	
Age					
≤15	89850	99.6	402	0.4	<.0001
16–64	285,095	95.1	14744	4.9	
65–74	81589	89.7	9405	10.3	
75–84	106912	85.7	17872	14.3	
≥85	44193	78.1	12416	21.9	
CCI score					
<1	141206	97.6	3483	2.4	<.0001
1–10	455338	90.7	46591	9.3	
11–20	11091	70	4762	30	
>21	4	57.1	3	42.9	
**Prehospital Level**					
Type of access to ED					
Direct access	469131	90.6	48440	9.4	<.0001
Transferred from other hospital	138508	95.6	6399	4.4	
Travel time to ED (min)					
<30	372100	92.2	31401	7.8	<.0001
30–60	101153	91.8	9009	8.2	
60–90	56689	91.7	5105	8.3	
>90	77817	89.4	9204	10.6	
**Hospital Level**					
Annual number of SED patients who visited ED					
1^st^ quartile (≥ 2701 cases)	358394	91.2	34457	8.8	<.0001
2^nd^ quartile (1801–2700 cases)	43420	90.1	4786	9.9	
3^rd^ quartile (601–1800 cases)	102978	92.9	7906	7.1	
4^th^ quartile (≤ 00 cases)	102847	93.0	7690	7.0	
Level of ED					
Regional emergency center	149626	90.9	14970	9.1	<.0001
Local emergency center (≥300 hospital beds)	256488	90.8	26141	9.2	
Local emergency center (<300 hospital beds)	19272	88.7	2445	11.3	
Local emergency agency (low volume)	77542	90.4	8241	9.6	
Unqualified emergency agency	104711	97.2	3042	2.8	
**HSA Level**					
Relevance Index					
≥71%	401270	91.9	35571	8.1	<.0001
51–70%	106623	91.5	9844	8.5	
31–50%	68 580	91.6	6328	8.4	
≤30%	31166	91.0	3096	9.0	
Number of regional and local emergency medical centers (per 100000 population)					
≥0.38	102582	92.6	8229	7.4	<.0001
0.26–0.38	367824	92.3	30597	7.7	
<0.26	137233	89.6	16013	10.4	
Existence of emergency medical center with 300 or more beds within HSA					
Yes (EC300)	575042	92.0	50302	8.0	<.0001
No (nEC300)	32597	87.8	4537	12.2	

Abbreviations: CCI, Charlson comorbidity index; ED, emergency department; HSA, health service area; SED, severe emergency disease. EC300, regional or local emergency center with 300 or more hospital beds located within an HSA; nEC300, no regional or local emergency center with 300 or more hospital beds located within an HSA.

**Figure 2:**
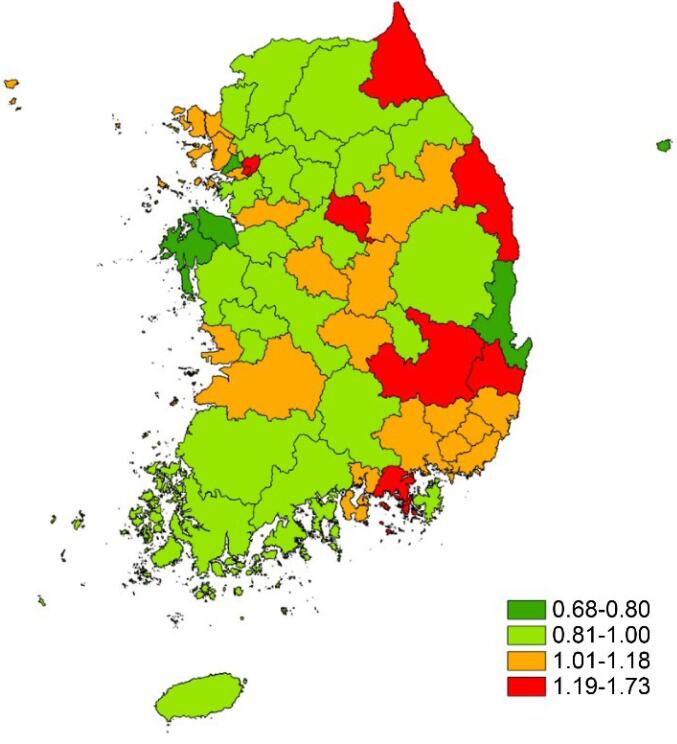
Distribution of Severe Emergency Disease Mortality Ratios Across 56 Health Service Areas.

 The mortality ratio according to 56 HSAs is displayed in Figure 2. When comparing HSAs, the highest SED mortality ratio among HSAs was approximately 2.5 times the lowest mortality ratio, a significant difference (Group 35 Dangjin-si 0.68 vs. Group 27 Sokcho-si 1.73; Table S4).

###  Main Analysis

Table 2 displays the results of the hierarchical logistic regression analysis using multilevel models. After adjustment for patient level, prehospital level, hospital level, and HSA level, nEC300 was significantly associated with increased 30-day mortality in SED patients (AOR: 1.33, 95% CI: 1.137-1.153).

**Table 2:** Logistic Regression Results for Key Variables and 30-Day Mortality

	**AOR**^a^	**95% CI**	***P*** ** Value**
Annual number of SED visits			
1^st^ quartile (≥2701 cases)	1		
2^nd^ quartile (1801–2700 cases)	1.181	(1.036–1.346)	.0126
3^rd^ quartile (601–1800 cases)	1.248	(1.136–1.372)	<.0001
4^th^ quartile (≤600 cases)	1.584	(1.372–1.828)	<.0001
Level of ED			
Regional emergency center	1		
Local emergency center (≥300 beds)	0.976	(0.915–1.041)	.4605
Local emergency center (<300 beds)	1.102	(1.070–1.135)	<.0001
Local emergency agency	1.115	(1.021–1.307)	.0222
Unqualified emergency agency	2.313	(1.926–2.778)	<.0001
Existence of regional and local emergency centers with 300 or more beds within HSA			
Yes (EC300)	1		
No (nEC300)	1.33	(1.137–1.153)	.003

Abbreviations: AOR, adjusted odds ratio; CI, confidence Interval; SED, severe emergency disease; ED, emergency department; HSA, health service area. ^a^Adjusted for patient level, prehospital level, hospital level, and HSA level variables. EC300, regional or local emergency center with 300 or more hospital beds located within an HSA; nEC300, no regional or local emergency center with 300 or more hospital beds located within an HSA.

 Patients who visited the ED at a hospital with a lower quartile of the annual number of SED visits were associated with greater 30-day mortality. Regarding ED level, local emergency centers with fewer than 300 hospital beds, local emergency agencies and unqualified emergency agencies had higher 30-day mortality rates than those of regional emergency centers. No statistically significant association of the odds of 30-day mortality was observed between regional emergency centers and local emergency centers with 300 or more beds (the results for all variables included in the model are displayed in Table S1).

## Discussion

 This study evaluated the association between the presence of an emergency center with 300 or more hospital beds in an HSA and the 30-day mortality of patients with SEDs in Korea using the NHID. The study results revealed that HSAs without regional or local emergency centers with 300 or more hospital beds (nEC300) had higher odds of 30-day mortality for patients with SEDs than HSAs with regional or local emergency centers with 300 or more hospital beds (EC300). The results indicate that a regulatory threshold value of 300 hospital beds could be utilized when evaluating emergency care resources over HSAs. In addition, the level of the ED and the volume of SED patients admitted to the hospital were associated with the 30-day mortality of patients with SEDs.

 Hospital volume is a known predictor of patient mortality,^21,27,28^ which can be explained by two main hypotheses: “practice-makes-perfect” and “selective referral pattern.”^29,30^ The “practice-makes-perfect” hypothesis holds that physicians and hospitals improve their skills by treating more patients. The “selective referral pattern” hypothesis holds that patients select physicians and hospitals based on their performance. Based on these two hypotheses, physicians and hospitals with high experience levels develop better outcomes. While previous studies have reported that regional disparities in resources could have an effect on the mortality of patients with cardiovascular disease, stroke or trauma,^4,31,32^ the association between hospital bed count and patient outcomes is controversial.^18-21,33^ For example, small hospitals (<400 beds) are not associated with lower quality except for heart attacks in the United Kingdom.^39^ In our study, a hospital bed count threshold of 300 was significantly associated with mortality, and an nEC300 was significantly associated with increased mortality.

 Thus, our study results further indicate that not only the number of EDs and hospitals but also the existence of a hospital and ED that exceeds a certain hospital bed capacity in an HSA has a positive effect on patient outcomes. The Korean government should focus not only on designating or establishing new emergency care facilities in vulnerable HSAs but also on promoting the establishment of hospitals and EDs that meet a minimum threshold (ie, regional or local emergency centers with 300 or more hospital beds) to ensure adequate care for SED patients.

 The 56 HSAs analyzed met the following criteria based on the hospital utilization patterns of the residents, as has been developed and utilized in previous studies: (1) minimum relevance index of 40%, (2) minimum background population of 150 000, and (3) transportation time within a region by car within 60 minutes.^25,34,35^ Disparities in healthcare resources regarding EC300 and the number of EDs were observed between HSAs (Table S4). In addition, the 30-day mortality of patients with SEDs varied between the HSAs (Table S4). Out of the 56 HSAs, 10 HSAs did not have any regional or local emergency centers with 300 or more hospital beds, whereas in the HSA including Seoul, the capital city of Korea, there were 31 regional or local emergency centers with 300 or more hospital beds. The variation in medical resources observed between HSAs is in line with previous studies regarding coronary artery bypass grafts, percutaneous coronary intervention rates and cesarean section rates.^34,35^ For example, the rate of coronary artery bypass graft placement was lower in the HSAs with nEC300.

###  Limitations

 Using the NHID, we adjusted our primary outcomes for patient-level, prehospital-level, hospital-level and HSA-level variables. However, we could not consider or adjust for patient severity other than the presence of an SED due to a lack of data to measure patient severity. As ED levels in Korea are designated and regulated by the national government based on the ED’s human resources, equipment, and availability of specialists,^36^ we adjusted for the level of the ED to account for hospital capacity to provide specialized treatment. However, due to limitations in our dataset, we could not adjust for hospital performance of specific treatments, such as percutaneous coronary intervention, coronary artery bypass grafting or surgery for specific diseases. We also could not adjust for the number of doctors due to the limitation of the claims database. We could not obtain information regarding the admission volume of individual hospitals, treatment volume for specific diseases, ED mortality or availability of human resources in our analysis.

 The adjustment for prehospital factors was limited to transfer status and travel time from the scene to the ED. The inclusion criterion for patients who were admitted from the ED for SED was that SEDs can be used for a diverse range of diseases ranging from cholecystitis to cardiac arrest and have different risks of 30-day mortality. Selection bias could also have occurred due to diagnostic input errors. Further subgroup analysis of specific disease categories is needed to account for these differences. In addition, our study method could not statistically estimate the minimum threshold bed count needed to ensure adequate care for SED patients but rather evaluated the predefined threshold of EC300.

## Conclusion

 nEC300 led to a higher risk of 30-day mortality in patients with SED than EC300 after adjusting for patient-level, prehospital-level, hospital-level and HSA-level variables. The results indicate that not only the number of EDs in each HSA but also the presence of EDs that have adequate receiving capacity (hospital beds) in each HSA are important for ensuring adequate patient outcomes. Policy-makers require evidence-based information for allocating healthcare resources, and studies on this topic are lacking because of the lack of consensus on the optimal hospital bed capacity for each HSA. Even though there are limited models of risk adjustment, the results of our study should be considered when allocating emergency medicine resources.

## Acknowledgment

 We thank Yoon Kim, Department of Health Policy and Management, Seoul National University College of Medicine, for his Innovative research concepts and Jung Eun Lee, College of Medicine, Catholic Kwandong University, for her writing assistance.

## Ethical issues

 The Institutional Review Board of Seoul National University approved this study (IRB No: 1704-131-848) and waived the need for participant consent because of the use of anonymized claim data.

## Competing interests

 Authors declare that they have no competing interests.

## Funding

 This study was supported by a research fund from NHI Service in Korea (https://www.nhis.or.kr/english/index.do; Grant number: HIRE 15-39). The funder did not play any role in the study design, data analysis, publication decision, or manuscript preparation. Mi Young Kwak is supported by NHI Service in Korea.

## Supplementary files

Supplementary file 1 contains Tables S1-S6.
