# Supplementary material for: Effect of the Presence of Emergency Departments With 300 or More Hospital Beds in Health Service Areas on 30-Day Mortality in Korea: A Nationwide Retrospective Cross-sectional Study
Source: Int J Health Policy Manag. 2024 May 12;13:8010. doi: 10.34172/ijhpm.2024.8010 (PMC11270603; doi:10.34172/ijhpm.2024.8010)
Supplement: Supplementary file 1 — contains Tables S1-S6. [file ijhpm-13-8010-s001.pdf]

Article title: Effect of the Presence of Emergency Departments With 300 or More Hospital Beds in Health Service Areas on 30-Day Mortality in Korea: A Nationwide Retrospective Cross-sectional Study

Journal name: International Journal of Health Policy and Management (IJHPM)

Authors' information: Stephen Gyung Won Lee[1], Haibin Bai[2], Joo Won Park[3], Seonhwa Lee[3], Mi Young Kwak[3]*, Won Mo Jang[4,5]*

[1]Department of Emergency Medicine, Seoul Metropolitan Government-Seoul National University Boramae Medical Center, Seoul, South Korea.

[2]Division of General Internal Medicine, Section of Biomedical Informatics and Data Science, School of Medicine, Johns Hopkins University, Baltimore, MD, USA.

[3]Center for Public Healthcare, National Medical Center, Seoul, South Korea.

[4]Department of Public Health and Community Medicine, Seoul Metropolitan Government-Seoul National University Boramae Medical Center, Seoul, South Korea.

[5]Department of Health Policy and Management, Seoul National University College of Medicine, Seoul, South Korea.

***Correspondence to:** Mi Young Kwak; Email: kmy805@gmail.com & Won Mo Jang; Email: thomasj@snu.ac.kr

Citation: Lee SGW, Bai H, Park JW, et al. Effect of the presence of emergency departments with 300 or more hospital beds in health service areas on 30-day mortality in Korea: a nationwide retrospective cross-sectional study. Int J Health Policy Manag. 2024;13:8010. doi:10.34172/ijhpm.2024.8010

Supplementary file 1

Table S1: Logistic regression results for all variables and 30-day mortality

Table S2: Definition of 28 severe emergency diseases

Table S3: Charlson Comorbidity Index Eligible Diseases and Weighted Values (Dr. Foster Rev. ICD-10)

Table S4: Number of regional or local emergency centers with 300 or more hospital beds and mortality ratio of 28 severe emergency diseases according to health service area

Table S5: Top 20 most frequent severe emergency diseases (SED) primary diagnosis according to study group

Table S6: Top 20 most frequent severe emergency diseases (SED) primary diagnosis causing mortality according to study group

Table S1. Logistic regression results for all variables and 30-day mortality

Variable		Odds Ratio (95% CI)	P-value
**Patient level**			
Sex	Female	Reference	
	Male	1.277 (1.245 – 1.310)	<.0001
Age	≤15	Reference	
	16~64	4.101 (3.083 – 4.422)	<.0001
	≥ 65	10.995 (9.902 – 12.208)	<.0001
Income level	≤ Top 20%	Reference	
	21-40%	1.021 (0.993 – 1.050)	0.1479
	41-60%	1.065 (1.031 – 1.100)	0.0002
	61-80%	1.109 (1.070 – 1.150)	<.0001
	81-100%	1.181 (1.155 – 1.207)	<.0001
Charlson Comorbidity Index score	<1	1	
	1 ~ 10	1.265 (1.207 – 1.325)	<.0001
	11 ~ 20	1.352 (1.274 – 1.434)	<.0001
	≥ 21	2.78 (2.611 – 2.960)	<.0001
**Pre-hospital level**			
Type of access to emergency department	Direct access	Reference	
	Transferred from other hospital	1.375 (1.249 – 1.512)	<.0001
Travel time to emergency department	< 30 min	Reference	
	30 ~ 60 min	1.143 (1.029 – 1.270)	0.0123
	61 ~ 90 min	1.157 (1.053 – 1.271)	0.0023
	>90 min	1.207 (1.095 – 1.332)	0.0002
**Hospital level**			
Annual number of severe emergency disease visit	1st quartile (≥ 2,701 cases)	Reference	
	2nd quartile (1,801-2,700 cases)	1.181 (1.036 – 1.346)	0.0126
	3rd quartile (601-1,800 cases)	1.248 (1.136 – 1.372)	<.0001
	4th quartile (≤ 600 cases)	1.584 (1.372 – 1.828)	<.0001
Level of emergency department	Regional center	Reference	
	Local center (≥300 beds)	0.976 (0.915 – 1.041)	0.4605
	Local center (<300 beds)	1.102 (1.070 – 1.135)	<.0001
	Local agency (low volume)	1.115 (1.021 – 1.307)	0.0222
	Unqualified institution	2.313 (1.926 – 2.778)	<.0001
**Health service area level**			
Relevance Index	≥ 71%	Reference	
	51~70%	0.961 (0.896 – 1.030)	0.2578
	31~50%	1.077 (0.971 – 1.195)	0.1605
	≤30%	1.044 (0.977 – 1.116)	0.1991
Number of regional and local emergency medical centers (per 100,000 population)	≥ 0.38	1.052 (1.012 – 1.101)	0.018
	0.26-0.38	Reference	
	< 0.26	1.029 (0.960 – 1.103)	0.4204
Existence of emergency medical center with 300 or more beds within health service area	Yes	Reference	
	No	1.33 (1.137 – 1.153)	0.003

Table S2. Definition of 28 severe emergency diseases

Classification of severe emergency diseases		ICD-10 code
1	Myocardial infarction	I210–I219
2	Cerebral infarction	I6300–I64
3	Cerebral hemorrhage	I610–I629
4	Subarachnoid hemorrhage	I600–I609
5	Severe trauma	
	Head injury	S0610–S0611, S0650–S0651, S0660–S0661, S0670–S0671, S0680–S0681, S0200, S0201, S02180, S02181, S0620, S0621, S0690, S0691
	Neck injury	S1500–S1508
	Chest injury	S2500–S2508, S26000–S226919, S27100–S27219, S280, S2730–S27319
	Pelvic fracture	S32820–S32891
	Abdominal injury	S3510–S3558, S357, S3590–S3598, S36100–S36112, S3670–S3671, S36800–S36818, S3770–S3771, S396
	Lower extremities injury	T0250–T0251, T790–T791, T794
	Nonfatal submersion	T751
	Asphyxiation	T71
	-	ICISS $\leq$ 0.90
6	Aortic dissection	I7101–I7109, I7110–I7119, I713, I715, I718
7	Gallbladder disease	K8000–K8011, K8030–K8041, K8051, K819, K830, K831
8	Surgical disease (intussusception/occlusion separate)	K352–K353, K631, K650–K659, K661
9	Gastrointestinal hemorrhage	I8500–I8501, I864, I983, K920–K922, K226, K2500, K2540, K2501, K2521, K2541, K2561, K260, K262, K264, K266, T181
10	Bronchial hemorrhage	R042, R048, R049, T1740–T1799
11	Intoxication (including CO)	T360–T659
12	Perinatal disease	O000–O009, O140–O159, O4200, O4201, O4209, O4210, O4211, O4219, O4220, O4221, O4229, O4290, O4291, O4299, O450–O459, O6000–O6039, O800–O809, O820–O829, O720–O723, O622
13	Premature baby/low birth weight infant	P0700–P0739, P220–P229, P240–P249, P360–P369, P520–P529, P590–P599
14	Severe burn	T3130–T3199, T2030–T2039, T2070–T2079, T213, T217
15	Status epilepticus	G410–G419
16	Encephalomeningitis	A830–A879, G000–G07

17	Septic embolism	A021, A227, A241, A267, A400–A409, A410–A414, A419, A427, B007, B377
18	Diabetic coma	E1000–E1018, E1100–E1118, E1300–E1318, E1400–E1418
19	Pulmonary embolism/DVT	I260, I269, I802
20	Arrhythmia	I441, I442, I450–I459, I472, I480–I489, I490, I495, I498, I499
21	ARDS/pulmonary edema	J80, J81, J850–J869, J9600–J9699
22	DIC	D65
23	Intussusception/occlusion	K561–K563, K565–K566
24	Dismemberment	S480–S489, S580–S589, S6800–S689, S780–S789, S880–S889, S980–S984, T050–T059, T060–T068, T116, T136
25	Acute renal failure	N170–N179
26	Eye-adaptive emergency	H3300–H3309, H3310–H332, H3330–H334, H3350–H3358, H340–H349, H400, H4010–H4019, H4020–H403, H404, H405, H406, H4080–H409, H420, H428
27	Post-resuscitation status	I460–I469
28	Urinary emergency	N44, N4500–N4502, N4590–N4592

ICD, International Classification of Disease.

Table S3. Charlson Comorbidity Index Eligible Diseases and Weighted Values (Dr. Foster Rev. ICD-10)

Condition No.	Condition Name	New Coding	New weight	Old weight
1	Acute myocardial infarction	I21, I22, I23, I252, I258	5	1
2	Cerebral vascular accident	G450, G451, G452, G454, G458, G459, G46, I60-I69	11	1
3	Congestive heart failure	I50	13	1
4	Connective tissue disorder	M05, M060, M063, M069, M32, M332, M34, M353	4	1
5	Dementia	F00, F01, F02, F03, F051	14	1
6	Diabetes	E101, E105, E106, E108, E109, E111, E115, E116, E118, E119, E131, E131, E136, E138, E139, E141, E145, E146, E148, E149	3	1
7	Liver disease	K702, K703, K717, K73, K74	8	1
8	Peptic ulcer	K25, K26, K27, K28	9	1
9	Peripheral vascular disease	I71, I739, I790, R02, Z958, Z959	6	1
10	Pulmonary disease	J40-J47, J60-J76	4	1
11	Cancer	C00-C76, C80-C97	8	2
12	Diabetes complications	E102, E103, E104, E107, E112, E113, E114, E117, E132, E133, E134, E137, E142, E143, E144, E147	-1	2
13	Paraplegia	G041, G81, G820, G821, G822	1	2
14	Renal disease	I12, I13, N01, N03, N052-N056, N072-N074, N18, N19, N25	10	2
15	Metastatic cancer	C77, C78, C79	14	3
16	Severe liver disease	K721, K729, K766, K767	18	3
17	HIV	B20, B21, B22, B23, B24	2	6

Table S4. Number of regional or local emergency centers with 300 or more hospital beds and mortality ratio of 28 severe emergency diseases according to health service **area**

**Health Service Area**		**Number of Regional or Local Emergency Center with 300 or More Hospital Beds**	**Mortality Ratio of 28 Severe Emergency Diseases**
Dangjin-si	Chungcheongnam-do (Dangjin-gun)	1	0.7
Siheung-si	Gyeonggi-do (Siheung-si)	0	0.8
Pohang-si	Gyeongsangbuk-do (Pohang-si, Yeongdeok-gun, Ulleung-gun)	2	0.8
Seosan-si	Chungcheongnam-do (Seosan-si, Taean-gun)	0	0.8
Geoje-si	Gyeongsangnam-do (Geoje-si)	0	0.8
Yeosu-si	Jeollanam-do (Yeosu-si)	1	0.8
Seongnam-si	Gyeonggi-do (Seongnam-si, Yongin-si, Gwangju-si)	5	0.9
Uijeongbu-si	Gyeonggi-do (Uijeongbu-si, Dongducheon-si, Yangju-si, Pocheon-si, Yeoncheon-gun),Gangwon-do (Cheorwon-gun)	1	0.9
Jeju	Jeju (Jeju-si, Seogwipo-si)	5	0.9
Guri-si	Gyeonggi-do (Guri-si, Namyangju-si, Yangpyeong-gun)	1	0.9
Nonsan-si	Chungcheongnam-do (Nonsan-si, Buyeo-gun)	1	0.9
Osan-si	Gyeonggi-do (Osan-si)	0	0.9
Gwangmyeong-si	Gyeonggi-do (Gwangmyeong-si)	1	0.9
Goyang-si	Gyeonggi-do (Goyang-si, Paju-si)	4	0.9
Hongseong-gun	Chungcheongnam-do (Boryeong-si, Cheongyang-gun, Hongseong-gun, Yesan-gun)	1	0.9
Suwon-si	Gyeonggi-do (Suwon-si, Hwaseong-si)	4	0.9
Icheon-si	Gyeonggi-do (Icheon-si, Yeoju-si)	0	0.9
Andong-si	Gyeongsangbuk-do (Andong-si, Yeongju-si, Uiseong-gun, Cheongsong-gun, Yeongyang-gun, Yecheon-gun, Bonghwa-gun)	2	0.9
Gwangju	Gwangju (all), Jeollabuk-do (Namwon-si, Sunchang-gun, Gochang-gun), Jeollanam-do (Naju-si, Damyang-gun, Gokseong-gun, Hwasun-gun, Hampyeong-gun, Yeonggwang-gun, Jangseong-gun)	6	1
Chuncheon-si	Gyeonggi-do (Gapyeong-gun), Gangwon-do(Chuncheon-si, Hongcheon-gun, Hwacheon-gun, Yanggu-gun)	2	1
Daejeon	Daejeon (all), sejong-si, Chungcheongbuk-do (Okcheon-gun), Chungcheongnam-do (Gongju-si, Gyeryong-si, Geumsan-gun), Jeollabuk-do (Muju-gun)	5	1

Jincheon-gun	Chungcheongbuk-do (Jincheon-gun, Goesan-gun, Eumseong-gun)	0	1
Mokpo-si	Jeollanam-do (Mokpo-si, Jangheung-gun, Gangjin-gun, Haenam-gun, Yeongam-gun, Muan-gun, Wando-gun, Jindo-gun, Sinan-gun)	2	1
Seoul	Seoul (all), Gyeonggi-do (Hanam-si)	31	1
Wonju-si	Gangwon-do (Wonju-si, Hoengseong-gun)	1	1
Gumi-si	Gyeongsangbuk-do (Gumi-si, Chilgok-gun)	2	1
Iksan-si	Jeollabuk-do (Iksan-si)	1	1
Gangneung-si	Gangwon-do (Gangneung-si, Pyeongchang-gun)	2	1
Suncheon-si	Jeollanam-do (Suncheon-si, Gwangyang-si, Gurye-gun, Goheung-gun, Boseong-gun), Gyeongsangnam-do (Hadong-gun)	1	1
Cheongju-si	Chungcheongbuk-do (Cheongju-si, Boeun-gun, Jeungpyeong-gun)	2	1
Cheonan-si	Chungcheongnam-do (Cheonan-si, Asan-si)	3	1
Jinju-si	Gyeongsangnam-do (Jinju-si, Sancheong-gun, Hamyang-gun, Geochang-gun, Hapcheon-gun)	2	1
Sacheon-si	Gyeongsangnam-do (Sacheon-si, Namhae-gun)	0	1
Bucheon-si	Gyeonggi-do (Bucheon-si)	3	1
Jeonju-si	Jeollabuk-do (Jeonju-si, Jeongeup-si, Gimje-si, Wanju-gun, Jinan-gun, Jangsu-gun, Imsil-gun, Buan-gun)	3	1
Incheon	Incheon (all)	8	1.1
Gimcheon-si	Gyeongsangbuk-do (Gimcheon-si), Chungcheongbuk-do (Yeongdong-gun)	1	1.1
Ulsan	Ulsan (all)	2	1.1
Gimpo-si	Gyeonggi-do (Gimpo-si)	1	1.1
Gunsan-si	Jeollabuk-do (Gunsan-si), Chungcheongnam-do (Seocheon-gun)	1	1.1
Mungyeong-si	Gyeongsangbuk-do (Sangju-si, Mungyeong-si)	1	1.1
Pyeongtaek-si	Gyeonggi-do (Pyeongtaek-si, Anseong-si)	1	1.1
Busan	Busan (all), Gyeongsangnam-do (Miryang-si)	8	1.1
Ansan-si	Gyeonggi-do (Ansan-si)	2	1.1
Yangsan-si	Gyeongsangnam-do (Yangsan-si)	1	1.1
Gimhae-si	Gyeongsangnam-do (Gimhae-si)	1	1.1
Jecheon-si	Gangwon-do (Yeongwol-gun, Jeongseon-gun), Chungcheongbuk-do (Jecheon-si, Danyang-gun)	1	1.2
Changwon-si	Gyeongsangnam-do (Changwon-si, Uiryeong-gun, Haman-gun, Changnyeong-gun)	3	1.2
Dague	Dague (all), Gyeongsangbuk-do (Yeongcheon-si, Gyeongsan-si, Gunwi-gun, Cheongdo-gun, Goryeong-gun, Seongju-gun)	6	1.2
Anyang-si	Gyeonggi-do (Anyang-si, Gwacheon-si, Uiwang-si)	2	1.2
Gunpo-si	Gyeonggi-do (Gunpo-si)	2	1.2
Donghae-si	Gangwon-do (Donghae-si, Taebaek-si, Samcheok-si), Gyeongsangbuk-do (Uljin-gun)	0	1.2

Goseong-gun	Gyeongsangnam-do (Tongyeong-si, Goseong-gun)	0	1.3
Gyeongju-si	Gyeongsangbuk-do (Gyeongju-si)	1	1.3
Chungju-si	Chungcheongbuk-do (Chungju-si)	1	1.7
Sokcho-si	Gangwon-do (Sokcho-si, Inje-gun, Goseong-gun, Yangyang-gun)	0	1.7

Table S5. Top 20 most frequent severe emergency diseases (SED) primary diagnosis according to study group

Study group	ICD code	Diagnosis	N	%
EC300	I639	Cerebral infarction, unspecified	40,445	6.5
	S0650	Traumatic subdural hemorrhage, without open intracranial wound	16,029	2.6
	J189	Pneumonia, unspecified	15,509	2.5
	K922	Gastrointestinal hemorrhage, unspecified	10,752	1.7
	I219	Acute myocardial infarction, unspecified	10,525	1.7
	I638	Other cerebral infarction	10,445	1.7
	I469	Cardiac arrest, unspecified	10,312	1.6
	A419	Sepsis, unspecified	10,248	1.6
	I214	Acute subendocardial myocardial infarction	9,742	1.6
	K8000	Calculus of gallbladder with acute cholecystitis, without mention of obstruction	8,152	1.3
	N179	Acute renal failure, unspecified	7,280	1.2
	I610	Intracerebral hemorrhage in hemisphere, subcortical	6,591	1.1
	N185	Chronic kidney disease, stage 5	5,980	1.0
	K921	Melaena	5,623	0.9
	I480	Paroxysmal atrial fibrillation	5,493	0.9
	R042	Hemoptysis	5,441	0.9
	I509	Heart failure, unspecified	5,153	0.8
	I6350	Middle cerebral artery	5,148	0.8
	A099	Gastroenteritis and colitis of unspecified origin	5,101	0.8
	I609	Subarachnoid hemorrhage, unspecified	4,901	0.8
nEC300	I639	Cerebral infarction, unspecified	2,844	7.7
	J189	Pneumonia, unspecified	1,457	3.9
	A419	Sepsis, unspecified	1,103	3.0
	S0650	Traumatic subdural hemorrhage, without open intracranial wound	950	2.6
	I638	Other cerebral infarction	877	2.4
	K922	Gastrointestinal hemorrhage, unspecified	624	1.7
	K8000	Calculus of gallbladder with acute cholecystitis, without mention of obstruction	527	1.4
	G819	Hemiplegia, unspecified	463	1.2
	I219	Acute myocardial infarction, unspecified	435	1.2
	K566	Other and unspecified intestinal obstruction	374	1.0
	I610	Intracerebral hemorrhage in hemisphere, subcortical	371	1.0
	N179	Acute renal failure, unspecified	371	1.0
	N390	Urinary tract infection, site not specified	354	1.0
	J690	Pneumonitis due to food and vomit	337	0.9
	K2501	Acute gastric ulcer with hemorrhage	322	0.9
	K8001	Calculus of gallbladder with acute cholecystitis, with obstruction	322	0.9
	J159	Bacterial pneumonia, unspecified	311	0.8
	N185	Chronic kidney disease, stage 5	311	0.8
	K650	Acute peritonitis	297	0.8
	G825	Tetraplegia, unspecified	288	0.8

Table S6. Top 20 most frequent severe emergency diseases (SED) primary diagnosis causing mortality according to study group

Study group	ICD code	Diagnosis	N	%
EC300	I469	Cardiac arrest, unspecified	7,198	14.3
	J189	Pneumonia, unspecified	2,831	5.6
	A419	Sepsis, unspecified	2,109	4.2
	I639	Cerebral infarction, unspecified	1,461	2.9
	C220	Liver cell carcinoma	1,261	2.5
	S0650	Traumatic subdural hemorrhage, without open intracranial wound	1,064	2.1
	I460	Cardiac arrest with successful resuscitation	972	1.9
	I219	Acute myocardial infarction, unspecified	915	1.8
	N179	Acute renal failure, unspecified	823	1.6
	J690	Pneumonitis due to food and vomit	783	1.6
	C3499	Malignant neoplasm of bronchus or lung, unspecified, unspecified side	676	1.3
	J9609	Acute respiratory failure, type unspecified	581	1.2
	N185	Chronic kidney disease, stage 5	552	1.1
	I609	Subarachnoid hemorrhage, unspecified	520	1.0
	I214	Acute subendocardial myocardial infarction	464	0.9
	I509	Heart failure, unspecified	457	0.9
	I610	Intracerebral hemorrhage in hemisphere, subcortical	426	0.8
	R572	Septic shock	420	0.8
	J80	Adult respiratory distress syndrome	411	0.8
	K922	Gastrointestinal hemorrhage, unspecified	409	0.8
nEC300	A419	Sepsis, unspecified	501	11.0
	J189	Pneumonia, unspecified	418	9.2
	I469	Cardiac arrest, unspecified	232	5.1
	I639	Cerebral infarction, unspecified	138	3.0
	J690	Pneumonitis due to food and vomit	131	2.9
	S0650	Traumatic subdural hemorrhage, without open intracranial wound	91	2.0
	I460	Cardiac arrest with successful resuscitation	88	1.9
	J159	Bacterial pneumonia, unspecified	86	1.9
	Z515	Palliative care	73	1.6
	J9609	Acute respiratory failure, type unspecified	72	1.6
	N179	Acute renal failure, unspecified	70	1.5
	I219	Acute myocardial infarction, unspecified	69	1.5
	C3499	Malignant neoplasm of bronchus or lung, unspecified, unspecified side	59	1.3
	I638	Other cerebral infarction	58	1.3
	J158	Other bacterial pneumonia	58	1.3
	I509	Heart failure, unspecified	42	1.1
	K922	Gastrointestinal hemorrhage, unspecified	41	1.1
	C220	Liver cell carcinoma	49	1.1
	N390	Urinary tract infection, site not specified	48	1.1
	I610	Intracerebral hemorrhage in hemisphere, subcortical	42	0.9
